# An RRx-001 Analogue With Potent Anti-NLRP3 Inflammasome Activity but Without High-Energy Nitro Functional Groups

**DOI:** 10.3389/fphar.2022.822833

**Published:** 2022-02-17

**Authors:** Hualong Lin, Mingyang Yang, Cong Li, Bolong Lin, Xianming Deng, Hongbin He, Rongbin Zhou

**Affiliations:** ^1^ Department of Geriatrics, First Affiliated Hospital of USTC, Division of Life Sciences and Medicine, University of Science and Technology of China, Hefei, China; ^2^ CAS Key Laboratory of Innate Immunity and Chronic Disease, School of Basic Medical Sciences, Division of Life Sciences and Medicine, University of Science and Technology of China, Hefei, China; ^3^ Chinese Academy of Sciences Centre for Excellence in Cell and Molecular Biology, University of Science and Technology of China, Hefei, China; ^4^ State Key Laboratory of Cellular Stress Biology, Innovation Center for Cell Signaling Network, School of Life Sciences, Xiamen University, Xiamen, China

**Keywords:** RRx-001 analogues, NLRP3 inflammasome, anti-inflammation, NLRP3-related inflammatory diseases, NLRP3 inhibitor

## Abstract

NLRP3 inflammasome is involved in the pathology of multiple human inflammatory diseases but there are still no clinically available medications targeting the NLRP3 inflammasome. We have previously identified RRx-001 as a highly selective and potent NLRP3 inhibitor, however, it contains high-energy nitro functional groups and may cause potential processing problems and generates highly toxic oxidants. Here, we show that compound 149-01, an RRx-001 analogue without high-energy nitro functional groups, is a potent, specific and covalent NLRP3 inhibitor. Mechanistically, 149-01 binds directly to cysteine 409 of NLRP3 to block the NEK7-NLRP3 interaction, thereby preventing NLRP3 inflammasome complex assembly and activation. Furthermore, treatment with 149-01 effectively alleviate the severity of several inflammatory diseases in mice, including lipopolysaccharide (LPS)-induced systemic inflammation, monosodium urate crystals (MSU)-induced peritonitis and experimental autoimmune encephalomyelitis (EAE). Thus, our results indicate that 149-01 is a potential lead for developing therapeutic agent for NLRP3-related inflammatory diseases.

## Introduction

The NLRP3 inflammasome is a multimeric protein complex consisted of innate immune sensor NLRP3 (NLR family, Pyrin domain containing 3), adaptor protein ASC and effector cysteine protease caspase-1, it plays a pivotal role in host defense against microbial infection and inflammation (Schroder and Tschopp, 2010; Davis et al., 2011). Unlike other sensor proteins, NLRP3 can be activated by a wide range of factors derived from microbes, the host and the environment (Broz and Dixit, 2016). Upon activation, NLRP3 recruits ASC, which then interacts with pro-caspase-1 to induce NLRP3 inflammasome complex assembly. This results in the cleavage and activation of caspase-1, which subsequently promotes the maturation and secretion of IL-1β and IL-18 and induces pyroptosis, a type of inflammatory cell death (Shi et al., 2015; Swanson et al., 2019). However, aberrant activation of NLRP3 inflammasome has been reported to promote the development of several human diseases, including gout, type 2 diabetes (T2D), inflammatory bowel disease (IBD), atherosclerosis, neurodegenerative diseases and others (Martinon et al., 2006; Bauer et al., 2010; Duewell et al., 2010; Masters et al., 2010; Wen et al., 2012; Heneka et al., 2013; Guo et al., 2015). Therefore, NLRP3 inflammasome is considered as a novel therapeutic target for these inflammatory diseases.

Currently, biologic agents targeting IL-1β are clinically used to treat NLRP3-driven diseases, including canakinumab (a neutralizing IL-1β monoclonal antibody), anakinra (a recombinant non-glycosylated IL-1 receptor antagonist), and rilonacept (a soluble decoy IL-1β receptor) (Dinarello et al., 2012). These agents have been proven to be effective in the treatment of Cryopyrin-associated periodic syndromes (CAPS) caused by NLRP3 gene mutations and have also been applied in clinical trials for other NLRP3-associated disorders (Dinarello et al., 2012; Dinarello and van der Meer, 2013). However, the NLRP3 inflammasome activation induces not only IL-1β production, but also drives pyroptosis and other proinflammatory cytokines production such as IL-18, which may also contribute to the pathogenesis of inflammatory diseases (Nowarski et al., 2015). Moreover, IL-1β can also be produced by other inflammasomes or in an inflammasome-independent manner (Davis et al., 2011; Netea et al., 2015), so blockage of IL-1β may lead to a higher risk of infection. Therefore, compared with biologic agents that target IL-1β, inhibitors that directly target NLRP3 inflammasome may be a better therapeutic option for NLRP3-related diseases.

In recent years, several small molecules, including MCC950, CY-09, OLT1177, oridonin, tranilast, INF39 and others, have been proposed to inhibit the NLRP3 inflammasome activation and exert remarkable therapeutic effects for NLRP3-associated disorders in animal models (Coll et al., 2015; Daniels et al., 2016; Jiang et al., 2017; He et al., 2018; Huang et al., 2018; Marchetti et al., 2018; Zhang et al., 2020; Shi et al., 2021). However, considering the safety, efficacy and specificity of these inhibitors, only a few have entered clinical trials, such as the MCC950-related agents (Inzomelid and IZD334), IFM-2427, OLT1177 (Mullard, 2019; Kluck et al., 2020). Although these agents hold promise for the treatment of NLRP3-related diseases, their clinical efficacy and safety remain to be further determined.

We previously described RRx-001 (1-bromoacetyl-3,3-dinitroazetidine), an antitumor agent in phase III clinical trials, as a novel potent NLRP3 inhibitor (Kim et al., 2016; Oronsky et al., 2019; Chen et al., 2021). However, RRx-001 is a small-molecule compound developed from the defense and aerospace industry and contains unique high-energy nitro functional groups (Oronsky et al., 2016). The gem-dinitroazetidine can decompose rapidly and may be sensitive to heat, impact, friction and electrostatic discharge (Ning et al., 2012; Straessler et al., 2012). Due to its inherent energy properties, the gem-dinitroazetidine can cause potential processing problems. In addition, these geminal dinitro-groups undergo decomposition under hypoxic conditions *in vivo*, producing the gaseous multifunctional free radical, nitric oxide (NO) (Fens et al., 2011; Oronsky et al., 2017). Further chemical interaction between NO and ROS (reactive oxygen species) can generate highly toxic oxidants, including nitrogen dioxide and peroxynitrite (van der Vliet et al., 2000; Oronsky et al., 2017). Moreover, our previous study has shown that removal of geminal dinitro-groups from RRx-001 resulted in a nearly 32-fold decrease in its inhibitory activity (Chen et al., 2021).

In this study, we tried to identify RRx-001 analogues with potent anti-NLRP3 inflammasome activity but without high-energy nitro functional groups. Our results showed that 149-01, an RRx-001 analogue, had comparable activity with RRx-001. Mechanistically, 149-01 blocked the NEK7-NLRP3 interaction by binding directly to cysteine 409 of NLRP3, thereby preventing the assembly of NLRP3 inflammasome complex. Moreover, 149-01 treatment effectively attenuated several NLRP3-related inflammatory diseases in mice, including LPS-induced systemic inflammation, MSU-induced peritonitis and EAE. Taken together, our findings demonstrate that 149-01 is active both *in vitro* and *in vivo*, suggesting that 149-01 is a potential lead for developing therapeutic agent for NLRP3-related human inflammatory diseases.

## Materials and Methods

### Mice

C57BL/6J mice were obtained from Shanghai SLAC Laboratory Animal Limited Liability Company (Shanghai, China). The generation of *Nlrp3* ^*−/−*^ mice were as previously described (Martinon et al., 2006). Mice were maintained in a SPF facility under a 12/12 h light/dark cycle (lights on at 07:00 and off at 19:00). All animal studies were performed in accordance with the guidelines of the Ethics Committee of University of Science and Technology of China.

### Reagents

ATP, Nigericin, MSU, and poly (A/T) were purchased from Sigma-Aldrich. Ultrapure LPS, Lipofextamine 2000, Pam3CSK4, MQAE, MitoSOX, MitoTracker, DAPI were acquired from Invitrogen. The Salmonella strain was a gift from Dr. Cai Zhang (Shandong University, Shandong, China). The C3 toxin was a gift from Dr. Tengchuan Jin (University of Science and Technology of China, Hefei, China). CY-09 was synthesized from Dr. Xianming Deng (Xiamen University, Xiamen, China). Protein G agarose (16-266) and anti-Flag (A2220) beads were bought from Millipore and Sigma-Aldrich, respectively. Anti-Flag (F2555) and anti-VSV (V4888) antibodies were from Sigma-Aldrich. The anti-mouse NLRP3 (AG-20B-0014) and the anti-mouse caspase-1 (AG-20B-0042) antibodies was from AdipoGen. The anti-mouse IL-1β antibody (AF-401-NA) was obtained from R&D Systems. The anti-*β*-actin antibody (66009-1-Ig) was from Proteintech Group. The anti-mouse ASC antibody (67824S) was from Cell Signaling Technology. The anti-mouse NEK7 antibody (ab133514) was from Abcam.

### Cell Preparation and Stimulation

BMDMs (bone marrow-derived macrophages) were collected from the bone marrow of 6–8 weeks old mice and cultured in DMEM supplemented with 10% FBS and 20 ng/ml murine M-CSF (Novoprotein). Human PBMCs (peripheral blood mononuclear cells) were obtained by Human Lymphocyte Separation Medium (catalog no. P8610-200, Solarbio) and cultured in RPMI 1640 medium containing 10% FBS.

To stimulate inflammasome activation, BMDMs (5×10^5^/well) or PBMCs (1×10^6^/well) were seeded into 12-well plates and cultured overnight. Cells were primed for 3 h in Opti-MEM with LPS (50 ng/ml) or Pam3CSK4 (400 ng/ml, for noncanonical inflammasome activation), followed by treatment with 149-01 for 30 min, and then stimulated with various inflammasome agonists: 3 µM nigericin (30 min); 150 μg/ml MSU (4 h); 2.5 mM ATP (30 min); S. typhimurium (4 h, multiplicity of infection (MOI)); 30 μM CL097 (1 h) or 0.5 μg/ml C3 toxin (6 h). 0.5 μg/ml poly (A/T) or 500 ng/ml LPS were transfected into BMDMs with Lipofectamine 2000 for 4 h or 16 h, respectively. The release of lactate dehydrogenase (LDH) was measured by LDH Cytotoxicity Assay Kit (Beyotime). Precipitated supernatants and cell lysates were further detected using western blotting.

*In vitro*, 149-01 was dissolved in DMSO, and the DMSO was used as vehicle control in all figures.

### ELISA

Cell culture supernatants and serum samples were analyzed for mouse IL-6, TNF-α or IL-1β and for human TNF-α or IL-1β with ELISA kit (all from R&D except human IL-1β from BD, 557953) according to the manufacturer’s instructions.

### Western Blotting

Sample buffer was added to resuspend precipitated supernatants or to lyse cells, and the cell lysates were transferred to 1.5 ml EP tube. All protein samples were further boiled at 100°C for 10 min and separated by 8% or 15% SDS–PAGE gels at 80 V for 0.5 h and at 120 V for 1 h. After that, proteins were transferred to PVDF membranes at 90 V for 1 h. The membranes were blocked with PBST containing 5% nonfat milk at room temperature for 1 h and then incubated with primary and conjugated secondary antibodies as described previously (Chen et al., 2021). After washing the membranes with PBST, protein bands were acquired after visualization by enhanced chemiluminescence.

### Detection of Intracellular K^+^ and Cl^−^ Levels

To determine intracellular K^+^ concentration, BMDMs (1×10^6^/well) seeded in 6-well plates were stimulated with nigericin to trigger NLRP3 inflammasome activation as described above. After that, cell culture supernatants were thoroughly removed and cells were lysed with ultrapure HNO_3_. Cell lysates were further boiled at 100°C for 30 min and then the precipitated products were dissolved with ddH_2_O. K^+^ concentrations were measured using PerkinElmer Optima 2000 DV spectrometer.

To determine intracellular Cl^−^ concentration, BMDMs seeded in 12-well plates were also stimulated to activate NLRP3 inflammasome. After stimulation, the culture supernatants were thoroughly discarded and cells were lysed with ddH_2_O (200 µl/well) at 37°C for 30 min. Cell lysates were transferred to 1.5 ml EP tube, centrifuged at 10,000×g for 5 min and then supernatants were mixed with MQAE (10 µM). The absorbance of the mixture was determined using BioTek Multi-Mode Microplate Readers (Synergy2). A control was set for each experiment to measure the extracellular residual Cl^−^ level after removal of the culture supernatant.

### Confocal Microscopy

BMDMs (2×10^5^/well) were seeded on coverslips (Thermo Fisher Scientific) and cultured overnight in 12-well plates. The next day, LPS-primed mouse BMDMs were stained with 5 μM MitoSOX or 50 nM MitoTracker Red and stimulated with nigericin. After stimulation, the culture supernatants were removed, and the cells were fixed *in situ* with 4% paraformaldehyde (PFA) in PBS for 15 min after washing by ice-cold PBS three times. Then, the cells were washed three times with PBST. Confocal microscopic analyses were utilized to detect mitochondrial damage by using a Zeiss LSM 700.

### Immunoprecipitation

To detect endogenous interaction, LPS-primed BMDMs were stimulated with nigericin in 6-well plates, then lysed in NP-40 lysis buffer containing protease inhibitor cocktail. The cell lysates were centrifuged at 10,000 rpm at 4°C for 10 min and the supernatants were then incubated with primary antibodies and Protein G Mag Sepharose overnight with rotation at 4°C. The protein-antibody complex was pulled down by protein G beads and determined using immunoblot analysis.

To detect exogenous interaction, plasmids were transfected into HEK-293T cells in 6-well plates via polyethylenimine (PEI). 24 h after transfection, cells were collected and lysed in NP-40 lysis buffer. After centrifugation, supernatants of cell lysates were co-incubated with anti-Flag antibody–coated beads. Then, immunocomplex proteins were pulled down by beads and analyzed using immunoblotting.

### ASC Oligomerization Assay

BMDMs (1×10^6^/well) were plated in 6-well plates and treated with nigericin. After stimulation, the cells were washed with ice-cold PBS and then lysed in NP-40 lysis buffer at 4°C for 30 min. The cell lysates were centrifuged at 330×*g* at 4°C for 10 min. The pellets were washed with ice-cold PBS three times and re-suspended in 500 μl PBS, then cross-linked with 2 mM disuccinimidyl suberate (DSS, Sangon Biotech, C100015) at room temperature for 30 min with rotation. The crosslinked pellets were collected after centrifugation at 330 *g* for 10 min and dissolved directly in 30 μl sample buffer, and analyzed using immunoblotting.

### Protein Expression and Purification

To purify human Flag-NLRP3 protein, human Flag-NLRP3 plasmid was transfected into HEK-293T cells via PEI and expressed for 48 h. The cells were then washed with ice-cold PBS and lysed in lysis buffer (50 mM HEPES, pH 7.4, 150 mM NaCl, and 0.4% CHAPS) at 4°C for 30 min. The cell lysates were centrifuged at 14,000 rpm at 4°C for 15 min. After centrifugation, supernatants of cell lysates were co-incubated with anti-Flag antibody–coated beads at 4°C for 2.5 h with rotation. The beads were collected after centrifugation and incubated with Flag peptide (Sigma-Aldrich, F3290) in elution buffer (50 mM HEPES, pH 7.4, 500 mM NaCl and 0.1% CHAPS) at 4°C for 90 min with rotation to elute Flag-NLRP3 protein. The eluents containing Flag-NLRP3 protein were collected and concentrated by ultrafiltration device (UFC910024; Merck Millipore) to remove proteins <100 kD. Meanwhile, 10 µl eluent was dissolved in sample buffer, separated by 8% SDS–PAGE gels, followed by detection using anti-Flag antibody.

To purify human GFP-NLRP3 protein, human His-GFP-NLRP3 plasmid was transfected into HEK-293T cells *via* PEI and expressed for 48 h. The cells were lysed in lysis buffer at 4°C for 30 min and sonicated. The supernatants of cell lysates were then collected by centrifuging at 14,000 rpm at 4°C for 15 min. The His-GFP-NLRP3 proteins were preliminarily isolated using nickel–nitrilotriacetic acid matrices (QIAGEN) at room temperature for 45 min, and the pulled down proteins were washed twice with lysis buffer. After that, proteins were eluted in elution buffer and the eluents were concentrated by ultrafiltration device to remove proteins <10 kD after filtration through a 0.45 μm syringe filter. Then, the final eluents were added to a Superdex 200 10/300 GL column (GE Healthcare) for further purification in 50 mM HEPES (pH 7.4) and 150 mM NaCl on an GE Healthcare AKTA purifier. The solutions containing His-GFP-NLRP3 protein were collected together and concentrated by ultrafiltration device to remove proteins <100 kD. Meanwhile, 10 µl solution was dissolved in sample buffer and analyzed using immunoblotting.

### NLRP3 ATPase Activity Assay

Purified human NLRP3 protein (1.4 ng/μl) was incubated with indicated doses of 149-01 or 1 µM CY-09 in reaction buffer at 37°C for 15 min and then ATP (25 μm, Ultra-Pure ATP) was added. After further incubation at 37°C for 40 min, the amount of ATP converted into ADP in the mixture was measured by luminescent ADP detection using ADP-Glo Kinase Assay kit (Promega, Madison, MI, United States). The data were presented as percentage of residual enzyme activity versus vehicle-treated enzyme activity.

### Microscale Thermophoresis Assay

A Monolith NT.115 instrument (NanoTemper Technologies) was used to measure the *K*_D_ value. Purified His-GFP-NLRP3 protein (200 nM) was incubated at room temperature with a range of doses of 149-01 (from 1 μM to 0.0169 nM) in assay buffer (50 mM HEPES, 10 mM MgCl_2_, 100 mM NaCl, pH 7.5, and 0.05% Tween 20) for 40 min. After the mixtures were loaded onto the NanoTemper glass capillaries, measurements were performed using 80% MST power and 100% LED power. *K*_D_ value calculations were done using the mass action equation through NanoTemper software from duplicate reads of an experiment.

### Drug Affinity Responsive Target Stability Assays

Assays were performed according to published protocols. BMDMs primed with LPS (3 h) or HEK-293T cells transfected with plasmid (24 h) were collected and lysed in NP-40 lysis buffer containing protease inhibitor cocktail. The cell lysates were centrifuged at 12,000×*g* at 4°C for 10 min and the supernatants were then measured by Pierce BCA Protein Assay Kit (Beyotime) to determine the protein concentration. After that, lysates (8 μg of protein lysate per reaction) were incubated at 4°C with indicated doses of 149-01 overnight with rotation. The mixtures were further incubated with protease pronase (25 ng of enzyme per μg of protein, Sigma) at room temperature for another 30 min. Then, the samples were dissolved directly in sample buffer and analyzed using immunoblotting.

### NLRP3 Reconstitution

*Nlrp3* ^*−/−*^ BMDMs were seeded into 12-well plates and cultured overnight. Then, cells were transduced with lentivirus (pLEX vector, Thermo Fisher) encoding mouse NLRP3 or their indicated mutant (C405A). After 6 h, the culture supernatant was removed and replaced with DMEM containing 10% FBS and 20 ng/ml murine M-CSF. After 48 h, BMDMs were stimulated with nigericin.

### Histological Analysis

Mouse spinal cords were fixed in 4% PFA at 4°C for 24 h, embedded in paraffin, and sectioned. Then, sections were stained with H&E or LFB according to standard procedures. Histological analysis for inflammatory cell infiltration and demyelination was performed with a Nikon ECL IPSE Ci biological microscope, and images were recorded using a Nikon DS-U3 color digital camera.

### Quantitative Real-Time PCR

Total RNA from tissues was extracted using TRIzol reagent (Takara). To synthesize cDNA, 800 ng of total RNA from each sample was reverse transcribed with Moloney murine leukemia virus (M-MLV) reverse transcriptase (Invitrogen). Then qPCR analysis was performed with SYBR Green premix (Takara Bio). All data were individually normalized to *Gapdh*. The primer sequences are shown as follows:

for mouse *Gapdh*: forward, GGT​GAA​GGT​CGG​TGT​GAA​CG; reverse, CTC​GCT​CCT​GGA​AGA​TGG​TG; for mouse *Il1b*: forward, TGC​CAC​CTT​TTG​ACA​GTG​ATG; reverse, AAG​GTC​CAC​GGG​AAA​GAC​AC; for mouse *Tnf*: forward, CGA​TGG​GTT​GTA​CCT​TGT​C; reverse, CGG​ACT​CCG​CAA​AGT​CTA​AG; for mouse *Il6*: forward, GTC​CTT​CCT​ACC​CCA​ATT​TCC; reverse, GCA​CTA​GGT​TTG​CCG​AGT​AGA.

### LPS-Induced Systemic Inflammation

8-week-old male mice were injected intraperitoneally with 5 mg/kg 149-01 or vehicle control (90% PBS plus 10% DMSO) for 30 min and then challenged with 20 mg/kg LPS. After 4 h, mice were sacrificed and the secretion of IL-1β and TNF-α in serum was detected by ELISA.

### MSU-Induced Peritonitis

8-week-old male mice pretreated with 5 mg/kg 149-01 or vehicle (90% PBS plus 10% DMSO) for 30 min were intraperitoneally injected with 1 mg MSU (dissolved in 0.5 ml PBS). After 6 h, mice were sacrificed and peritoneal cavities were washed with 10 ml ice-cold PBS. The proportion of polymorph nuclear neutrophils was detected by flow cytometry and the number of peritoneal exudate cells was counted. In addition, the secretion of IL-1β in lavage fluid or serum was also measured using ELISA.

### Induction and Assessment of EAE

8-week-old male mice were subcutaneously immunized with 300 μg MOG_35-55_ peptide emulsified in CFA supplemented with 5 mg/ml (0.5 mg/mouse) of heat-killed Mycobacterium tuberculosis. Mice were intravenously injected with 150 ng pertussis toxin on days 0 and 2.149-01 (5 mg/kg) was administered intraperitoneally to mice on the day of induction and every 2 days thereafter. Control mice were given vehicle (90% PBS plus 10% DMSO) at the same time points. Clinical disease scores were recorded according to the following standard scale: 0, no abnormalities; 1, limp tail or waddling gait with tail tonicity; 2, wobbly gait; 3, hind limb paralysis; 4, hind limb and forelimb paralysis; 5, death. Mononuclear cells isolated from both the brain and spinal cord using 30% Percoll separation were analyzed by flow cytometry.

### Statistical Analyses

All data are presented as mean ± s.e.m. Statistical analysis was carried out with one-way ANOVA (GraphPad Software) for data in all figures (except Figure 7 and Supplementary Figure S7) or with unpaired Student’s t-tests (GraphPad Software) for data in Figure 7 and Supplementary Figure S7, and differences were considered significant at *p*<.05. No values were excluded.

## Results

### 149-01 Blocks NLRP3 Inflammasome Activation

**FIGURE 1:**
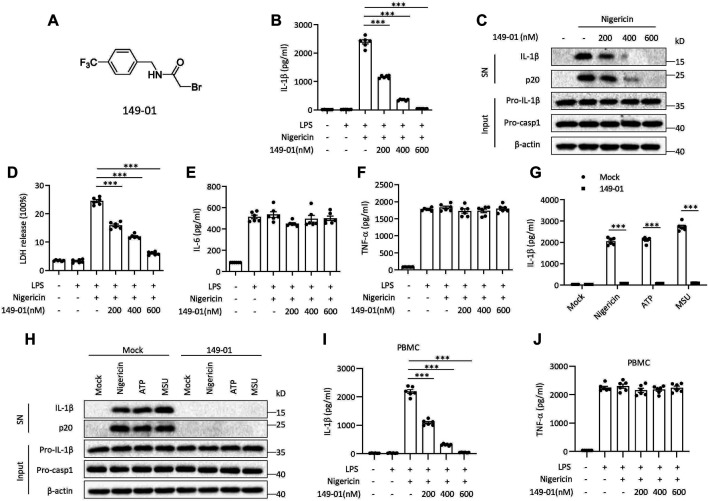
Identification of 149-01 as a highly potent inhibitor for NLRP3 inflammasome. **(A)** 149-01 structure. **(B–F)** BMDMs were first primed with LPS for 3 h, then pretreated with indicated doses of 149-01 (200-600 nM) for 30 min, lastly stimulated with nigericin. **(B)** IL-1β releases in supernatants were detected by ELISA. **(C)** Active IL-1β and p20 (cleaved caspase-1) in supernatants (SN) and pro-IL-1β, pro-caspase-1 and *β*-actin in cell lysates (Input) were measured by western blot. **(D)** The release of LDH in supernatants. **(E)** IL-6 and **(F)** TNF-α secretion levels in supernatants were determined by ELISA. **(G,H)** BMDMs were first primed with LPS, then treated with or without 149-01 (600 nM) for 30 min, lastly stimulated with nigericin, ATP or MSU. **(G)** IL-1β releases in supernatants were detected by ELISA. **(H)** Active IL-1β and p20 in supernatants and pro-IL-1β, pro-caspase-1 and *β*-actin in cell lysates were measured by western blot. **(I,J)** PBMCs were first primed with LPS for 3 h, then pretreated with indicated doses of 149-01 for 30 min, lastly stimulated with nigericin. **(I)** IL-1β and **(J)** TNF-α secretion levels in supernatants were determined by ELISA. Data are obtained from three independent experiments, each with two biological replicates and are expressed as mean ± s. e.m (*n* = 6) **(B,D–G,I,J)**, or are representative of three independent experiments **(C,H)**. One-way ANOVA was applied to calculate statistical significance: ***p < .001.

Several RRx-001 analogues were screened for their NLRP3 inhibitory activity in Supplementary Figure S1A and their chemical structure were depicted in Supplementary Table S1. In the screening results of RRx-001 analogues, 149-01 showed comparable NLRP3 inhibitory activity with RRx-001. To further evaluate the inhibitory effect of 149-01 on NLRP3 inflammasome activation, mouse bone marrow-derived macrophages (BMDMs) were first primed with LPS, then pretreated with 149-01 for 30 min, lastly stimulated with the NLRP3 stimulus nigericin. We found that 149-01 dose-dependently inhibited IL-1β release and caspase-1 activation at the doses of 200-600 nM (Figures 1A–C). Consistently, 149-01 also suppressed nigericin-induced release of lactate dehydrogenase (LDH) (Figure 1D), but LPS-dependent IL-6 and TNF-α secretion was not affected (Figures 1E,F), which suggests that 149-01 inhibits NLRP3-dependent cytokine production and pyroptosis. To ascertain whether 149-01 only blocked NLRP3 activation induced by nigericin, we tested the effects of 149-01 on NLRP3 activation triggered by two additional agonists, ATP and MSU. We observed that 149-01 inhibited IL-1β release and caspase-1 activation triggered by both NLRP3 agonists (Figures 1G,H). In addition, 149-01 also dose-dependently suppressed intracellular LPS-induced noncanonical NLRP3 inflammasome activation (Supplementary Figures S2A,B) but did not reduce LDH release (Supplementary Figure S2C). As previously reported, intracellular LPS induced LDH release was independent of NLRP3 (Coll et al., 2015). These results demonstrate that 149-01 displays potent and broad inhibitory effects on both canonical and non-canonical NLRP3 activation.

We next investigated whether 149-01 was effective for human cells. We isolated human peripheral blood mononuclear cells (PBMCs), treated LPS-primed cells with 149-01 for 30 min and then activated NLRP3 inflammasome with nigericin. We observed that 149-01 treatment reduced IL-1β release in a dose-dependent manner but not TNF-α production (Figures 1I,J). Furthermore, LPS alone induced alternative NLRP3 inflammasome activation was also suppressed by 149-01 in PBMCs (Supplementary Figures S2D,E). These results together indicate that 149-01 can prevent NLRP3 inflammasome activation in both mouse and human cells.

Because RRx-001 is reported to inhibit pro-IL-1β expression in macrophages (Yu et al., 2019; Chen et al., 2021), we then tested whether 149-01 influenced LPS-induced priming phase of NLRP3 inflammasome activation. We found that at concentrations of 200-600 nM, 149-01 treatment before or after LPS stimulation did not affect LPS-induced pro-IL-1β, NLRP3 production and IL-6, TNF-α secretion (Supplementary Figures S3A–C), indicating that 149-01 does not influence LPS-induced priming phase at concentrations that blocks NLRP3 activation. Although at higher concentrations of 1–4 μM, 149-01 treatment before LPS stimulation markedly inhibited LPS-induced pro-IL-1β and IL-6 production but did not affect NLRP3 or TNF-α expression (Supplementary Figures S4A–C), 149-01 showed approximately 9 times inhibitory activity against IL-1β release than that against IL-6 production (Supplementary Figures S4D,E). Besides, higher concentrations (one to four μM) of 149-01 treatment after LPS stimulation did not affect LPS-induced pro-IL-1β, NLRP3 production and IL-6, TNF-α secretion (Supplementary Figures S4A–C).

### 149-01 Does Not Affect AIM2, NLRC4, or Pyrin Inflammasomes Activation

**FIGURE 2:**
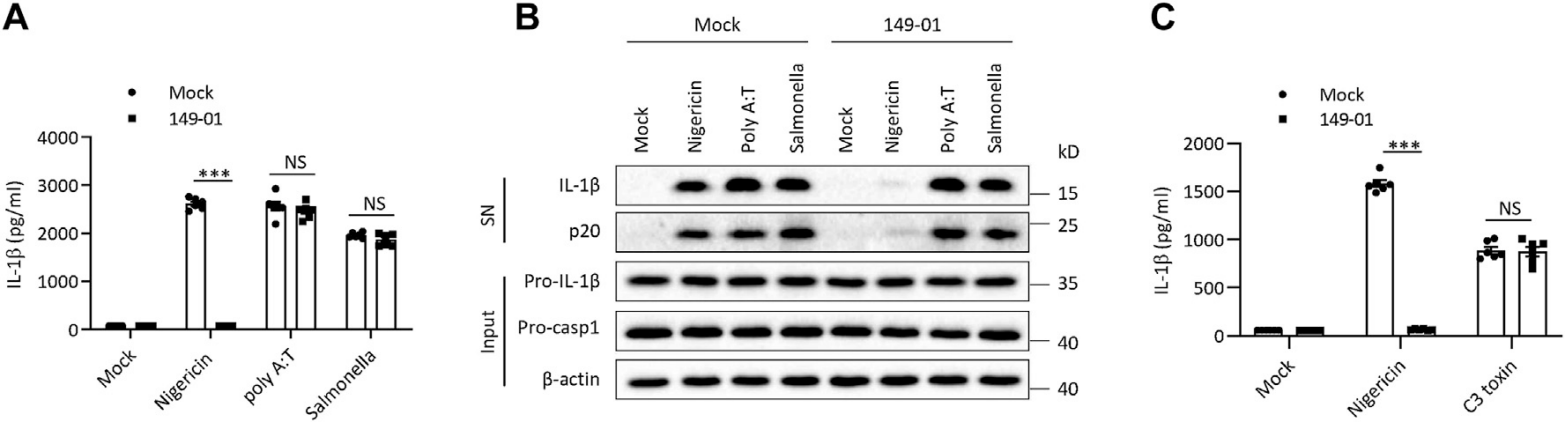
149-01 does not affect the activation of AIM2, NLRC4, or Pyrin inflammasomes. **(A–C)** BMDMs were first primed with LPS, then treated with or without 149-01 (600 nM) for 30 min, lastly stimulated with nigericin, poly (A/T), Salmonella or C3 toxin. **(A,C)** IL-1β releases in supernatants were detected by ELISA. **(B)** Active IL-1β and p20 in supernatants and pro-IL-1β, pro-caspase-1 and *β*-actin in cell lysates were measured by western blot. Data are obtained from three independent experiments, each with two biological replicates and are expressed as mean ± s. e.m (*n* = 6) **(A,C)**, or are representative of three independent experiments **(B)**. One-way ANOVA was applied to calculate statistical significance: ***p < .001, NS, not significant.

Besides NLRP3, several other sensors were also proposed to trigger inflammasome complex assembly (Xu et al., 2014; Broz and Dixit, 2016). To explore the specificity of 149-01’s inhibitory activity against NLRP3 inflammasome, we examined whether 149-01 could suppress the activation of three other well-characterized inflammasome. Murine BMDMs were first primed with LPS, then transfected with double-stranded DNA analog poly (A/T) to induce the AIM2 (Absent In Melanoma 2) inflammasome activation or infected with S. typhimurium to induce the NLRC4 (NLR Family CARD Domain Containing 4) inflammasome activation or stimulated with C3 toxin to induce the Pyrin inflammasome activation. In contrast to its potent inhibitory effects on NLRP3 inflammasome activation, 149-01 could not inhibit IL-1β release or caspase-1 activation following AIM2 or NLRC4 inflammasome activation (Figures 2A,B). In addition, 149-01 also could not attenuate IL-1β release triggered by Pyrin inflammasome activation (Figure 2C). Collectively, our data show that 149-01 selectively suppresses NLRP3 inflammasome activation.

### 149-01 Suppresses the Assembly of NLRP3 Inflammasome by Preventing the NEK7-NLRP3 Interaction

Next, we sought to identify the precise mechanism by which 149-01 prevents NLRP3 inflammasome activation. Potassium (K^+^) efflux and chloride (Cl^−^) efflux are proposed to be upstream events required for NLRP3 activation (Munoz-Planillo et al., 2013; Tang et al., 2017). To determine whether 149-01 abrogates NLRP3 activation by affecting these upstream events, we tested intracellular levels of K^+^ and Cl^−^ after nigericin stimulation. We found that pretreatment with 149-01 in BMDMs did not prevent the nigericin-induced decrease in intracellular K^+^ and Cl^−^ (Supplementary Figures S5A,B). Consistent with this, we also observed that 149-01 could dose-dependently suppress NLRP3 inflammasome activation induced by CL097, which is a K^+^ efflux-independent NLRP3 agonist (Supplementary Figure S5C) (Gross et al., 2016). In addition, mitochondrial damage is considered to be another upstream event required for NLRP3 activation (Zhou et al., 2011; Gurung et al., 2015). We next detected the mitochondrial status of BMDMs after nigericin stimulation and observed abnormal mitochondrial fission, clustering, and enhanced ROS production. However, pretreatment with 149-01 did not significantly affect these processes (Supplementary Figure S5D). Taken together, our findings indicate that 149-01 blocks NLRP3 activation by acting downstream of K^+^ efflux, Cl^−^ efflux and mitochondrial damage.

**FIGURE 3:**
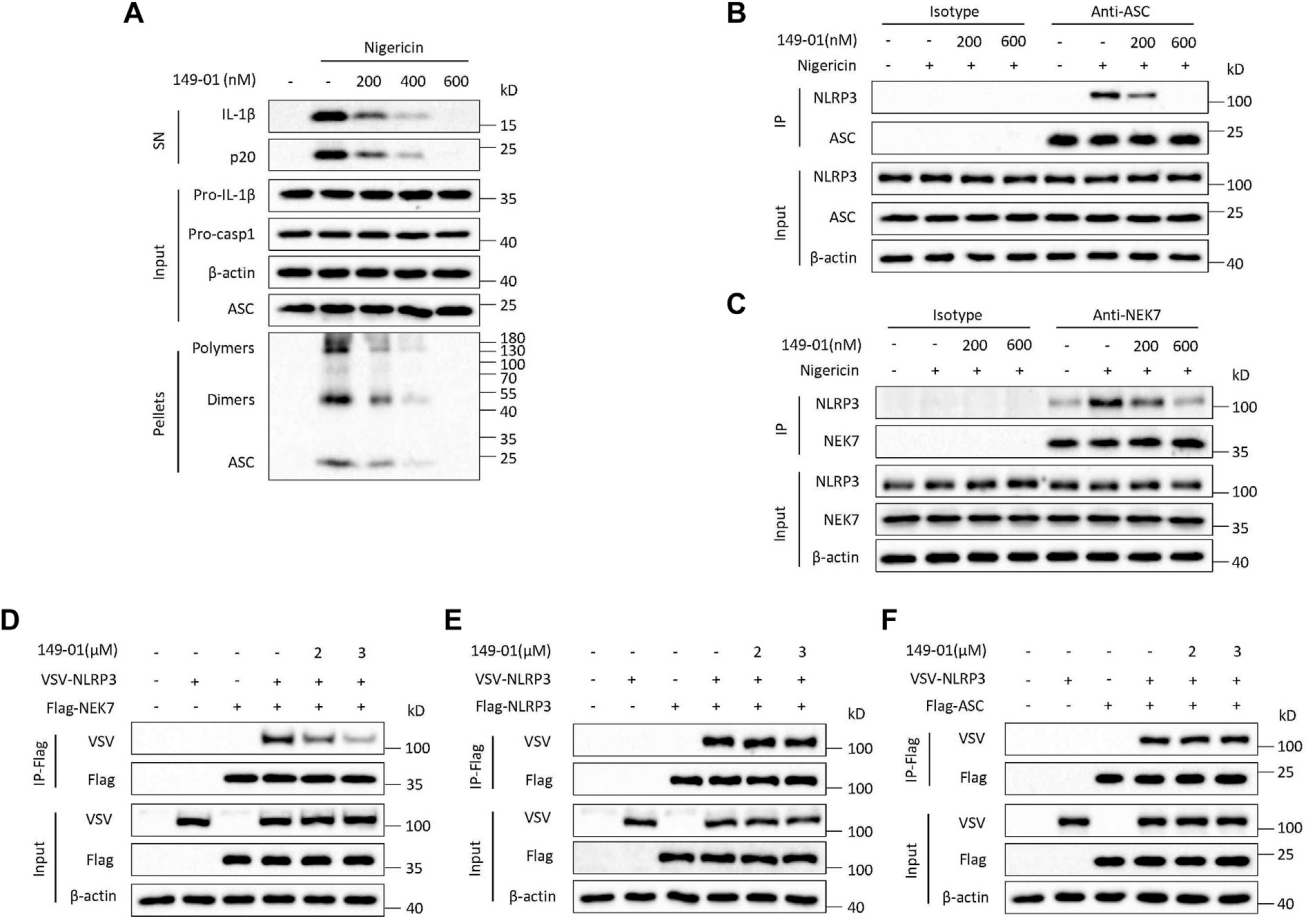
149-01 blocks the assembly of NLRP3 inflammasome by preventing the interaction between NEK7 and NLRP3. **(A)** Supernatants, cell lysates and DSS-crosslinked pellets from LPS-primed BMDMs treated with indicated doses of 149-01 for 30 min before nigericin stimulation were analyzed by western blot. **(B)** The endogenous NLRP3-ASC interaction in LPS-primed BMDMs treated with indicated doses of 149-01 for 30 min before nigericin stimulation was detected by immunoprecipitation (IP) and western blot. **(C)** The endogenous NEK7-NLRP3 interaction in LPS-primed BMDMs treated with indicated doses of 149-01 for 30 min before nigericin stimulation was detected by IP and western blot. **(D)** The NEK7–NLRP3 interaction in HEK-293T cells transfected with Flag-NEK7, VSV-NLRP3 plasmids and treated with indicated doses of 149-01 was evaluated by IP and western blot. **(E)** The NLRP3–NLRP3 interaction in HEK-293T cells transfected with Flag-NLRP3, VSV-NLRP3 plasmids and treated with indicated doses of 149-01 was evaluated by IP and western blot. **(F)** The NLRP3–ASC interaction in HEK-293T cells transfected with Flag-ASC, VSV-NLRP3 plasmids and treated with indicated doses of 149-01 was evaluated by IP and western blot. Data are representative of three independent experiments.

We then tested whether 149-01 could block NLRP3 activation by influencing NLRP3 inflammasome complex assembly. A critical step for NLRP3 inflammasome complex assembly and subsequent caspase-1 activation is ASC oligomerization (Lu et al., 2014; Dick et al., 2016), which can be detected after chemical cross-linking followed by western blotting. Consistent with inhibition of IL-1β release and caspase-1 activation, 149-01 markedly suppressed ASC oligomerization induced by nigericin at the doses of 200-600 nM without affecting ASC expression in cell lysates (Figure 3A), indicating that 149-01 prevents NLRP3 activation by acting upstream of ASC oligomerization. Next, we examined by coimmunoprecipitation whether 149-01 could block NLRP3-ASC interaction, which is essential for ASC recruitment and subsequent ASC oligomerization (Swanson et al., 2019). We observed that 149-01 dramatically attenuated the interaction between endogenous NLRP3 and ASC in nigericin stimulated BMDMs (Figure 3B). Moreover, we investigated if 149-01 influenced NEK7–NLRP3 interaction, which is imperative for NLRP3 oligomerization and subsequent NLRP3-ASC complex formation (He et al., 2016; Shi et al., 2016). We found that 149-01 substantially abolished the nigericin-mediated endogenous NEK7-NLRP3 interaction (Figure 3C). Collectively, these findings reveal that 149-01 may prevents NLRP3 inflammasome complex assembly by blocking the NEK7-NLRP3 interaction.

To validate this hypothesis, we overexpressed Flag-NEK7 and VSV-NLRP3 in HEK-293T cells, and explored by coimmunoprecipitation whether 149-01 could inhibit direct NEK7-NLRP3 interaction. The results showed that 149-01 treatment blocked the direct NEK7-NLRP3 interaction (Figure 3D). In contrast, 149-01 treatment had no impact on the direct NLRP3-NLRP3 or NLRP3-ASC interaction (Figures 3E,F). Therefore, these results suggest that 149-01 inhibits NLRP3 inflammasome activation by abrogating the direct NEK7-NLRP3 interaction, rather than by affecting NLRP3 oligomerization or subsequent NLRP3-ASC complex formation. Consistent with this, we found that 149-01 did not impact the NLRP3 ATPase activity (Supplementary Figure S6A), which is critical for the NLRP3 self-oligomerization (Duncan et al., 2007). In the same experiment, the known NLRP3 inflammasome inhibitor CY-09 was used as a positive control (Jiang et al., 2017). In addition, NEK7 has also been reported to act downstream of NEK9 and contribute to mitotic progression (Haq et al., 2015). However, 149-01 treatment did not affect the interaction between NEK7 and NEK9 (Supplementary Figure S6B).

### 149-01 Binds Directly to Cys409 of NLRP3

**FIGURE 4:**
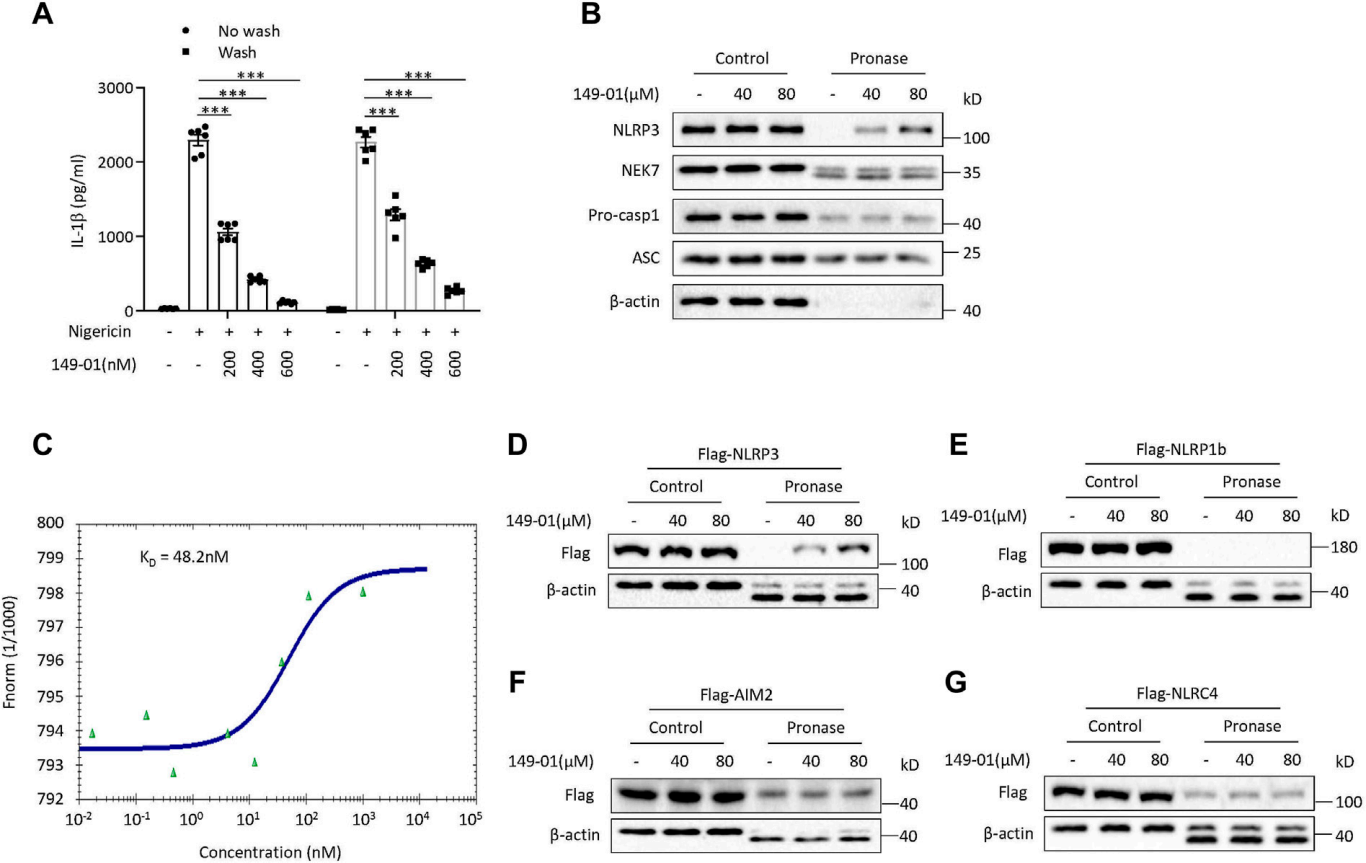
149-01 irreversibly, directly, and specifically binds to NLRP3. **(A)** IL-1β releases in supernatants from LPS-primed BMDMs treated with indicated doses of 149-01 for 15 min, washed three times before nigericin stimulation were detected by ELISA. **(B)** LPS-primed BMDMs cell lysates incubated overnight with indicated doses of 149-01 before pronase digestion were detected by western blot using antibodies for NLRP3, NEK7, pro-caspase-1, ASC and *β*-actin. **(C)** Binding affinity of 149-01 to purified GFP-NLRP3 protein was analyzed by MST. HEK-293T cells transfected with Flag-tagged NLRP3 **(D)**, NLRP1b **(E)**, AIM2 **(F)**, or NLRC4 **(G)** were lysed and the cell lysates incubated overnight with indicated doses of 149-01 before pronase digestion were detected by western blot. Data are obtained from three independent experiments, each with two biological replicates and are expressed as mean ± s. e.m (*n* = 6) **(A)**, or are representative of three independent experiments **(B–G)**. One-way ANOVA was applied to calculate statistical significance: ***p < .001.

We next determined whether the activation of NLRP3 inflammasome inhibited by 149-01 was reversible. LPS-primed mouse BMDMs were first treated with 149-01 for 15 min, then washed every 5 min for three times to remove unbound 149-01, and lastly stimulated with nigericin. We observed that nigericin-induced IL-1β release remained inhibited after removal of unbound 149-01, suggesting that 149-01’s inhibitory effect on NLRP3 activation is essentially irreversible (Figure 4A). In contrast, the inhibitory effects of CY-09 on NLRP3 activation is reversible (Supplementary Figure S6C). Together these data suggest that 149-01 may bind its target protein in a covalent manner.

Based on the result that 149-01 blocks the direct NEK7-NLRP3 interaction, we inferred that 149-01 may prevents NLRP3 inflammasome complex assembly by directly targeting NEK7 or NLRP3. To confirm this conjecture, we performed the drug affinity responsive target stability (DARTS) assay (Lomenick et al., 2009; Coll et al., 2019), which exploits the target protein’s reduced sensitivity to protease when it interacts with small molecules. We incubated the LPS-primed BMDMs cell lysates with different concentrations of 149-01, then digested with the protease pronase, and observed that 149-01 dose-dependently reduced proteolysis of NLRP3 by pronase (Figure 4B), suggesting that 149-01 directly interacts with NLRP3. However, incubation with 149-01 did not affect pronase mediated degradation of other NLRP3 inflammasome components, including NEK7, pro-casp1 and ASC (Figure 4B), indicating that 149-01 specifically targets NLRP3. To further validate the direct binding of 149-01 to NLRP3, we employed microscale thermophoresis (MST) assay to quantify the direct interaction between 149-01 and purified GFP-NLRP3. The data revealed that 149-01 interacted with purified GFP-NLRP3 with an equilibrium dissociation constant (K_D_) of 48.2 nM (Figure 4C). Therefore, our results reveal that 149-01 binds directly to NLRP3 with high affinity. To investigate whether 149-01 interacted with other inflammasome sensors, we overexpressed flag-tagged NLRP3, NLRP1b, AIM2 or NLRC4 in HEK-293T cells and then performed DARTS experiments. The results showed that 149-01 protected only NLRP3 but not NLRP1b, AIM2 or NLRC4 from pronase-induced proteolysis in a dose-dependent manner (Figures 4D–G).

**FIGURE 5:**
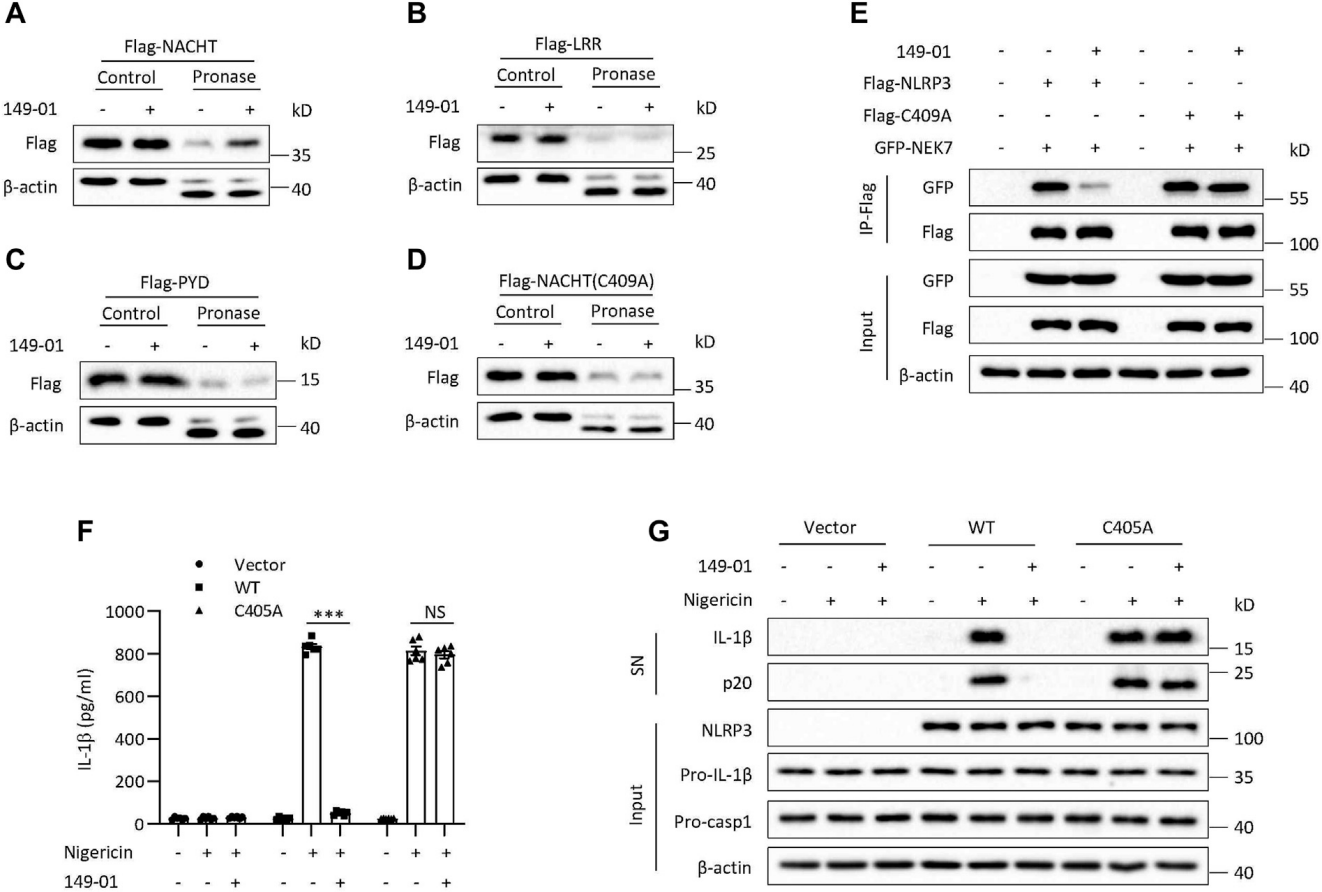
149-01 targets Cys409 of NLRP3. HEK-293T cells transfected with Flag-tagged NACHT **(A)**, LRR **(B)**, PYD **(C)**, or NACHT (C409A) **(D)** were lysed and the cell lysates incubated overnight with or without 149-01 (80 μM) before pronase digestion were detected by western blot. **(E)** The interactions between NEK7 and WT or mutant NLRP3 in HEK-293T cells treated with or without 149-01 (3 μM) were evaluated by IP and western blot. **(F,G)** *Nlrp3* ^*−/−*^ BMDMs reconstituted with mouse WT or mutant NLRP3 were first primed with LPS, then treated with or without 149-01 (600 nM) for 30 min, lastly stimulated with nigericin. **(F)** IL-1β releases in supernatants were detected by ELISA. **(G)** Active IL-1β and p20 in supernatants and NLRP3, pro-IL-1β, pro-caspase-1 and *β*-actin in cell lysates were measured by western blot. Data are obtained from three independent experiments, each with two biological replicates and are expressed as mean ± s. e.m (*n* = 6) **(F)**, or are representative of three independent experiments **(A–E,G)**. One-way ANOVA was applied to calculate statistical significance: ***p < .001, NS, not significant.

We next overexpressed three functional domains of NLRP3 in HEK-293T cells, NACHT, LRR and PYD, respectively, to explore which domain is responsible for interacting with 149-01. We found that only the NACHT domain but not the LRR or PYD domain could be protected by 149-01 from pronase-mediated proteolysis (Figures 5A–C). These results together demonstrate that 149-01 directly and specifically targets the NACHT domain of NLRP3. Next, we sought to determine the 149-01-binding residue within the NLRP3 NACHT domain. Since RRx-001 has been reported to bind to cysteine 409 (Chen et al., 2021), we tested whether 149-01 also targeted the C409 site of the NACHT domain. Flag-tagged the NACHT domain with the C409A mutation was overexpressed in HEK-293T cells and then performed DARTS assay. We observed that the C409A mutation abrogated the interaction between 149-01 and the NACHT domain (Figure 5D). Furthermore, the C409A mutation had no effect on the NEK7-NLRP3 interaction, but abolished 149-01’s inhibitory effect on this interaction (Figure 5E), suggesting that 149-01 binds directly to C409 of NLRP3. We then reconstituted *NLRP3* ^*−/−*^ BMDMs with mouse WT NLRP3 or mutant NLRP3 (C405A, corresponding to human NLRP3-C409A) and found that nigericin-induced NLRP3 activation was blocked by 149-01 in cells reconstituted with WT NLRP3 but remained intact in cells reconstituted with mutant NLRP3 (Figures 5F,G). Collectively, our results suggest that 149-01 binds directly to cysteine 409 of NLRP3 and then prevents the NEK7-NLRP3 interaction and ultimately inhibits NLRP3 inflammasome activation.

### 149-01 Suppresses NLRP3 Inflammasome *in vivo*

**FIGURE 6:**
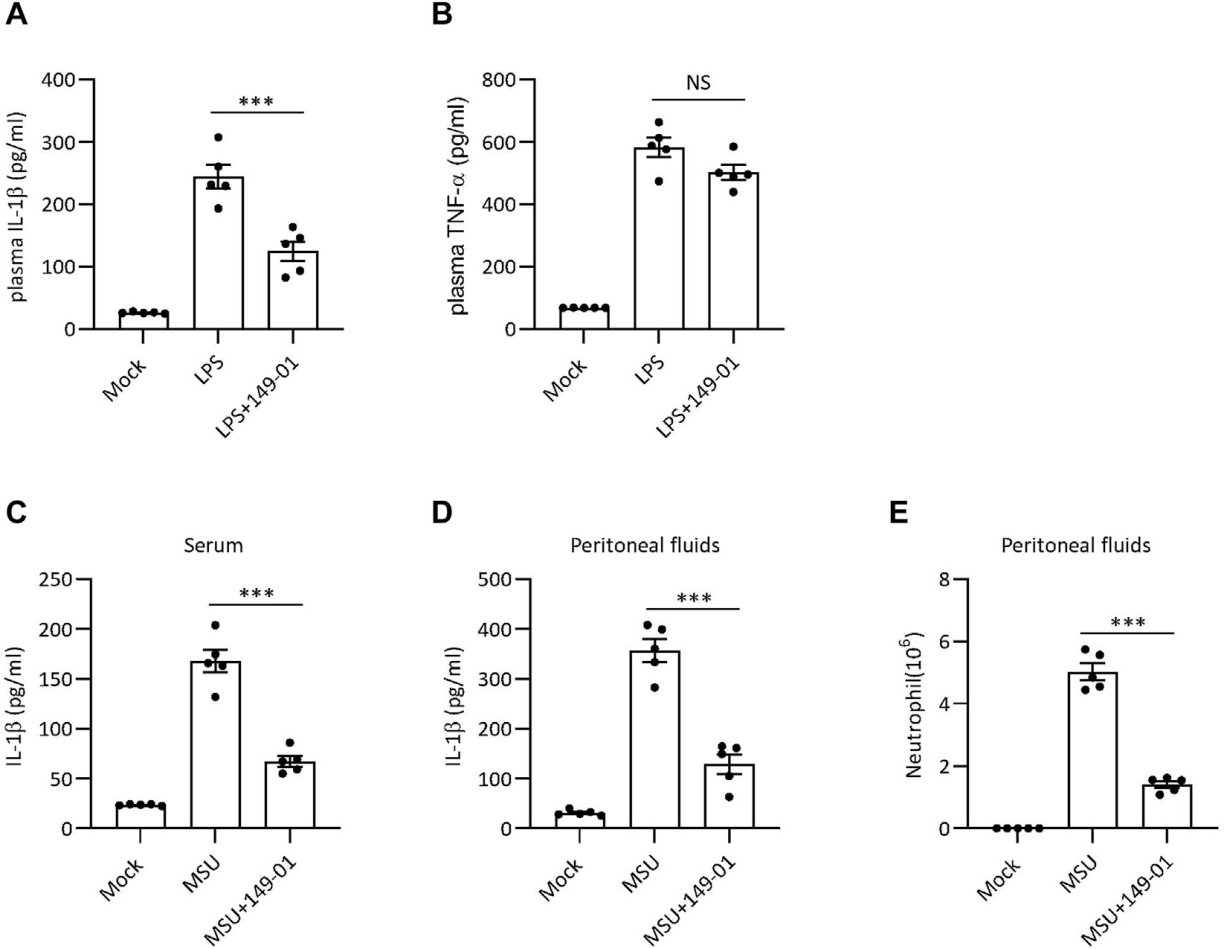
149-01 alleviates LPS-induced systemic inflammation and MSU-induced peritonitis in mice. **(A,B)** Serum IL-1β **(A)** and TNF-α **(B)** levels in mice intraperitoneally injected with 149-01 (5 mg/kg) or vehicle before challenge with LPS (20 mg/kg) were measured by ELISA. **(C–E)** Mice were intraperitoneally injected with 149-01 (5 mg/kg) or vehicle before challenge with MSU (1 mg/mouse). IL-1β in the serum **(C)** or peritoneal cavity **(D)** were measured by ELISA. **(E)** Neutrophil numbers in the peritoneal cavity were determined by FACS. Data are expressed as mean ± s. e.m (*n* = 5). One-way ANOVA was applied to calculate statistical significance: ***p < .001, NS, not significant.

Since 149-01 disrupts NLRP3 inflammasome activation *in vitro*, we next tested the therapeutic efficacy of 149-01 in NLRP3-dependent septic shock disease model, in which intraperitoneal injection of LPS could elicit NLRP3-driven IL-1β production (He et al., 2013). Mice were injected with 149-01 intraperitoneally before challenge with LPS and were evaluated 4 h later. The data revealed that pretreatment with 149-01 markedly downregulated serum IL-1β level without considerably decreasing NLRP3-independent TNF-α production in serum (Figures 6A,B). These findings indicate that 149-01 suppresses LPS-induced NLRP3-related systemic inflammation *in vivo*.

MSU crystal-induced peritonitis is another well-established NLRP3-dependent acute inflammatory model characterized by IL-1β secretion and massive neutrophil infiltration in peritoneal cavity (Martinon et al., 2006). To investigate the inhibitory activity of 149-01 in peritonitis model, mice were pretreated with 149-01 before intraperitoneal injection of MSU. As expected, pretreatment with 149-01 efficiently inhibited IL-1β secretion and neutrophil recruitment (Figures 6C–E). Our results suggest that 149-01 significantly alleviates MSU-induced peritonitis in mice.

**FIGURE 7:**
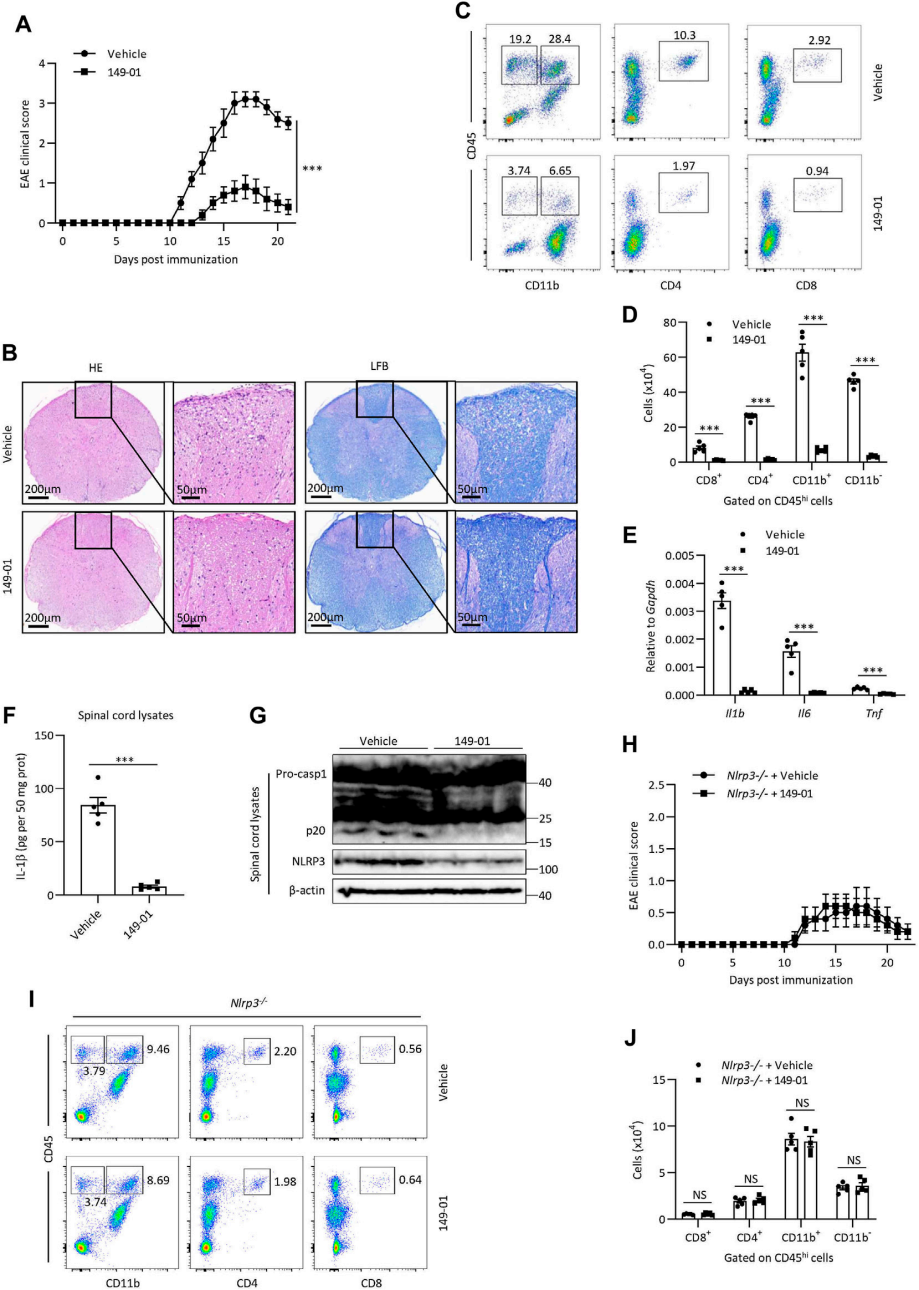
149-01 attenuates the progression of EAE *via* inhibition of NLRP3 inflammasome. **(A–G)** Mice were intraperitoneally injected with 149-01 (5 mg/kg) or vehicle on the day of EAE induction and every 2 days thereafter. **(A)** EAE clinical scores. **(B)** Representative spinal cord sections were stained with H&E or Luxol fast blue (LFB) to visualize inflammatory cell infiltration or demyelination at day 14 after EAE induction. **(C,D)** The percentages of representative plots **(C)** and total numbers **(D)** of infiltrated CD8^+^, CD4^+^, CD11b^+^ and CD11b^−^ cells gated on CD45^hi^ cell populations in the CNS were determined by FACS at day 14 after EAE induction. **(E)** The expression of indicated inflammatory cytokines in the CNS were detected by qPCR at day 14 after EAE induction. **(F,G)** The protein levels of IL-1β, caspase-1 and NLRP3 in the spinal cords were detected by ELISA **(F)** or western blot **(G)** at day 14 after EAE induction. **(H–J)** *Nlrp*3^**−/−**^ mice were intraperitoneally injected with 149-01 (5 mg/kg) or vehicle on the day of EAE induction and every 2 days thereafter. **(H)** EAE clinical scores. **(I,J)** The percentages of representative plots **(I)** and total numbers **(J)** of infiltrated CD8^+^, CD4^+^, CD11b^+^ and CD11b^−^ cells gated on CD45^hi^ cell populations in the CNS were determined by FACS at day 14 after EAE induction. Data are expressed as mean ± s. e.m (*n* = 5) **(A,D–F,H,J)** or are representative of five mice **(B,C,G,I)**. Unpaired Student’s t-tests were applied to calculate statistical significance: ***p < .001, NS, not significant.

The experimental autoimmune encephalomyelitis (EAE) mouse model, which effectively simulates human multiple sclerosis disease, is characterized by inflammation and demyelination. Considering that NLRP3 inflammasome acts a key role in the pathogenesis of EAE and exacerbates neuroinflammation (Sutton et al., 2006; Gris et al., 2010; Lalor et al., 2011), we then assessed whether 149-01 could alleviate the severity of EAE *via* targeting NLRP3 inflammasome. We induced EAE by subcutaneous immunizing mice with myelin oligodendrocyte glycoprotein (MOG) peptide (MOG_35–55_) and recorded clinical disease scores on a daily basis. Compared with the vehicle-treated group, treatment of mice with 149-01 improved their clinical behaviors (Figure 7A). Consistent with this phenotype, in mice 14 days after EAE induction, HE staining revealed that 149-01 treatment ameliorated inflammatory cell infiltration in the spinal cord and LFB staining showed that 149-01 treatment reduced spinal cord demyelination (Figure 7B). In addition, the proportions and absolute numbers of infiltrated T cells (CD8^+^ and CD4^+^), total lymphocytes (CD11b^−^ CD45^hi^), activated resident microglial cells and myeloid cells (CD11b^+^ CD45^hi^) gated on CD45^hi^ cell populations in the CNS were markedly reduced in 149-01-treated mice (Figures 7C,D). Further investigation of inflammation in EAE mice showed that 149-01 treatment significantly inhibited the expression of inflammatory cytokines in the CNS, including IL-1β, IL-6 and TNF-α (Figure 7E). Furthermore, 149-01 treatment significantly reduced protein levels for IL-1β, caspase-1 and NLRP3 in the spinal cords of EAE mice (Figures 7F,G). Collectively, these findings suggest that the disease progression of EAE can be attenuated effectively by 149-01 administration. To confirm that 149-01 mitigate EAE severity through suppression of NLRP3 inflammasome, we induced EAE in *NLRP3* knockout mice. We found that treatment of *NLRP3* ^*−/−*^ mice with 149-01 did not affect the clinical disease scores of EAE (Figure 7H) or the proportions and numbers of infiltrated immune cells in the CNS (Figures 7I,J). We also evaluated the safety profile of 149-01 in naive mice. Mice were intraperitoneally injected with 5 mg/kg 149-01 or vehicle control once a day for 4 weeks, and the results showed that 149-01 treatment did not affect the metabolic parameters and serum chemistry of naive mice (Supplementary Figure S7). Taken together, our results demonstrate that 149-01 is active *in vivo* and can mitigate the progression of inflammatory diseases by targeting NLRP3 inflammasome.

## Discussion

In this study, we identify compound 149-01, an RRx-001 analogue, as a potent, specific and covalent NLRP3 inhibitor, which can effectively inhibit the NLRP3 inflammasome activation both *in vitro* and *in vivo*. Our study suggests that 149-01 may be a useful small molecule tool to investigate the mechanism of NLRP3 inflammasome activation and a potential lead for developing therapeutic agent for NLRP3-driven inflammatory diseases.

Our results showed that the IC50 (half-maximal inhibitory concentration) value of 149-01 inhibiting IL-1β release is 182.8 nM, indicating that its inhibitory activity is stronger than most of the reported NLRP3 inhibitors, and 149-01 exhibits comparable inhibitory activity against NLRP3 inflammasome as RRx-001 (Chen et al., 2021). At the doses of 200-600 nM, 149-01 dose-dependently suppressed canonical and non-canonical NLRP3 activation in mouse cells and canonical and alternative NLRP3 activation in human cells. Meanwhile, 149-01 had no effect on the activation of AIM2, NLRC4, or Pyrin inflammasome, nor did it affect the priming phase of NLRP3 inflammasome at this concentration. These results demonstrate that 149-01 displays potent, broad and specific inhibitory effects on NLRP3 activation. We also observed that 149-01 treatment before LPS stimulation significantly inhibited LPS-induced pro-IL-1β and IL-6 production at higher doses of 1–4 μM, and the concentration required to inhibit IL-6 release is ∼9 times higher than that required to inhibit IL-1β release, suggesting that 149-01 may have better therapeutic activity in NLRP3-related inflammatory disorders than in other inflammatory disorders. However, the specific mechanism by which high doses of 149-01 inhibits LPS-induced pro-IL-1β and IL-6 production and its possible effects remain unclear. One possibility is that high doses of 149-01 also affect the function of other proteins, and high doses of 149-01 affect pro-IL-1β and IL-6 production by targeting other proteins.

Mechanistically, our data indicated that 149-01 does not block NLRP3 inflammasome activation by affecting upstream events, including K^+^ efflux, Cl^−^ efflux and mitochondrial damage (Zhou et al., 2011; Munoz-Planillo et al., 2013; Gurung et al., 2015; Tang et al., 2017), suggesting that 149-01 may directly affect the assembly of NLRP3 inflammasome complex. Indeed, 149-01 blocked the interaction between NEK7 and NLRP3 by irreversibly targeting cysteine 409 of NLRP3, ultimately inhibiting the NLRP3 inflammasome assembly and activation. However, according to the determined cryo-electron microscopy structure of human NLRP3-NEK7 complex (Sharif et al., 2019), we found that cysteine 409 is not at the interface of NLRP3-NEK7 interaction. We speculate that 149-01 binding to the C409 site of NLRP3 may cause a conformational change of NLRP3, resulting in the blockage of the NLRP3-NEK7 interaction, but how 149-01 binds to the cysteine 409 of NLRP3 and how this combination changes the conformation of NLRP3 remain to be further studied.

Our data revealed that 149-01 effectively alleviate LPS-induced systemic inflammation, MSU-induced peritonitis and EAE at a dose of 5 mg/kg and its therapeutic effect is dependent on NLRP3. In previous study, the dosage of 10 mg/kg RRx-001, was used in the treatment of multiple NLRP3-dependent disease models (Chen et al., 2021), indicating that 149-01 may possess *in vivo* therapeutic efficacy comparable to or better than RRx-001. Notably, in EAE mice model, 149-01 treatment significantly inhibited the expression of inflammatory cytokines in the CNS such as IL-6 and TNF-α in contrast to *in vitro* results. We suggested that the reduced inflammatory cytokines in the CNS may result from 1) 149-01 treatment alleviated the infiltration of inflammatory cell that produce these inflammatory cytokines; and 2) 149-01 treatment inhibited the expression of IL-1β which could mediate these inflammatory cytokines production via IL-1 receptors (Mori et al., 2011; Dinarello, 2018). Since RRx-001 has been shown to have a high safety profile in clinical trials (Reid et al., 2015; Oronsky et al., 2017; Oronsky et al., 2019), its analogue, 149-01, may have a higher potential to enter clinical trials.

Compared with RRx-001, the structure of 149-01 does not contain high-energy nitro functional groups, which avoids the side effects of NO production on the treatment of NLRP3-related diseases. In addition, 149-01 also avoids potential processing problems, for example, 1) the gem-dinitroazetidine can decompose rapidly, resulting in decreased yields; and 2) the gem-dinitroazetidine is sensitive to heat, impact, friction and electrostatic discharge, causing potential safety hazards (Straessler et al., 2012).

It has been proposed that inhibitors directly targeting NLRP3 have certain advantages over biologic agents targeting IL-1β in the treatment of NLRP3-related inflammatory disorders (Jiang et al., 2017), but there are still no clinically available therapeutic drugs. Although at least some small molecules have entered clinical trials, their clinical efficacy and safety remain to be further confirmed (Mullard, 2019; Kluck et al., 2020).

Considering the potent and specific inhibitory effect of 149-01 on NLRP3 inflammasome both *in vitro* and *in vivo*, and the high safety of RRx-001 in clinical trials (Reid et al., 2015; Oronsky et al., 2017; Oronsky et al., 2019), we consider that 149-01, as an analogue of RRx-001, has promising therapeutic potential for NLRP3-related diseases. However, as mentioned above, there are some concerns remain to be further investigated, which may limit the application of 149-01 as a NLRP3 inhibitor.

## Data Availability

The original contributions presented in the study are included in the article/Supplementary Materials, further inquiries can be directed to the corresponding authors.
